# Occupational burnout mediates the association between team climate and nurses’ safety behaviours: a multicentre cross-sectional study

**DOI:** 10.3389/fpubh.2026.1744333

**Published:** 2026-03-13

**Authors:** Fang Xu, Chenxi Li, Liqun Zhu, Yingying Wang

**Affiliations:** 1 Nursing Department, Affiliated Hospital of Jiangsu University, Zhenjiang, Jiangsu, China; 2 Department of Emergency, Yijishan Hospital of Wannan Medical College, Wuhu, Anhui, China

**Keywords:** mediation analysis, nurses, occupational burnout, patient safety, safety behaviours, team climate

## Abstract

**Background:**

In contemporary healthcare, nurses are pivotal to patient safety, and their behaviours are integral to care quality and adverse event prevention. However, escalating organisational complexity and sustained workload demands have made burnout prevalent among nurses, with demonstrable adverse effects on performance and patient safety. Although prior research indicates that a positive team climate fosters desirable nursing behaviours, the extent to which it promotes safety behaviours by alleviating burnout remains unclear. Clarifying whether and how burnout mediates the association between team climate and nurses’ safety behaviours would refine theoretical accounts of behavioural formation in nursing practice and provide actionable evidence to guide organisational strategies and managerial interventions that optimise workforce well-being and enhance the quality and safety of care.

**Objective:**

To examine whether occupational burnout mediates the association between team climate and nurses’ safety behaviours.

**Methods:**

Between March and April 2025, we conducted a multicentre cross-sectional survey using convenience sampling among registered nurses from three tertiary grade A hospitals in Anhui and Jiangsu provinces, China. Data were collected using self-administered questionnaires comprising a sociodemographic form, the Nurses’ Safety Behaviours Scale, the Maslach Burnout Inventory–General Survey (MBI–GS), and the short form of the Team Climate Inventory (TCI).

**Results:**

Among 925 nurses, the mean (SD) safety behaviours score was 52.89 (9.14). Safety behaviours correlated positively with team climate and negatively with overall burnout and each burnout dimension (all *p* < 0.05). In regression analyses, burnout negatively predicted safety behaviours, whereas team climate positively predicted them; the model explained 45.6% of the variance (*R*^2^ = 0.456). Mediation analysis indicated a partial mediation effect of burnout in the association between team climate and safety behaviours: the total effect was *B* = 0.599 (95% CI 0.552–0.646; *p* < 0.001), and the indirect effect via burnout was *B* = 0.084 (95% CI 0.058–0.114; *p* < 0.001).

**Conclusion:**

Occupational burnout undermines nurses’ safety behaviours, whereas a favourable team climate strengthens them. Moreover, team climate indirectly enhances safety behaviours by mitigating burnout. These findings have practical implications for nursing management and training. Interventions should prioritise mitigating burnout and cultivating a positive team climate to strengthen nurses’ safety behaviours.

## 1 Background

Patient safety is a fundamental aspect of modern clinical care and a critical indicator of healthcare quality. As global public health challenges intensify, patient safety has become a strategic priority across healthcare systems worldwide (1). Adverse events (AEs) pose a significant threat to patient safety: they prolong hospital stays, increase costs, and can result in serious sequelae, including disability and death (2, 3). Evidence suggests that approximately 46% of AEs are preventable with effective interventions (4), with preventability estimates varying from 17 to 76.5% across different settings (5). Within this landscape, nurses are indispensable to patient safety. Through central roles in aseptic technique, patient identification, and medication management, nurses exert a direct influence on the prevention of AEs (6, 7).

Nurses’ safety behaviours refer to actions undertaken in clinical practice to prevent patient harm and enhance patient safety (8). Typical behaviours include verifying patient identifiers, performing hand hygiene, using appropriate personal protective equipment, and adhering rigorously to standardised workflows—practices that significantly reduce the risk of AEs (9, 10). Determinants of nurses’ safety behaviours are multifaceted, encompassing both individual- and organisational-level factors, including years of experience, professional rank, educational attainment, team dynamics, organisational and safety culture, and personality traits (11–13).

Amid increasing clinical complexity, nurses face escalating workload and psychological stressors, leading to widespread burnout, which undermines performance and job satisfaction (14). Burnout—characterised by emotional exhaustion, depersonalisation, and reduced personal accomplishment—is prevalent in the nursing workforce (15, 16). Globally, approximately 30.7% of nurses experience burnout, which erodes job satisfaction and productivity, jeopardising patient safety (15). Burnout is associated with safety-related failures, including care omissions, medication errors, suboptimal infection prevention and control, and a weakened safety culture (15, 17).

Team climate is an organisational-level psychosocial construct that reflects team members’ shared perceptions of policies, practices, and interpersonal dynamics within the work environment (18). A positive team climate fosters collaboration and trust, thereby improving performance and safety-related behavioural outcomes. In nursing—a high-intensity, high-responsibility field—a favourable climate provides socio-emotional support and resource exchange, which may mitigate burnout. According to the Job Demands–Resources (JD–R) model, job resources and job demands influence burnout through a motivational (gain) pathway and a health-impairment (strain) pathway, respectively (19). As a key job resource, team climate may directly enhance nurses’ safety behaviours and indirectly promote them by alleviating burnout arising from high job demands. Although prior studies have examined associations between burnout and nurses’ safety behaviours, the direct link between team climate and safety behaviours—and the underlying mechanisms—remains underexplored. Grounded in the JD–R model, this study investigates how team climate shapes nurses’ safety behaviours, focusing on the mediating role of burnout, to inform efforts to optimise the work environment, reduce burnout, and ultimately improve care quality and patient safety.

## 2 Hypotheses

Based on the preceding rationale and theoretical framework, we hypothesise that:

*H1*: Team climate is positively associated with nurses' safety behaviours.

*H2*: Occupational burnout mediates the association between team climate and nurses' safety behaviours.

### 2.1 Study design

We conducted a multicentre, cross-sectional survey between March and April 2025 in three tertiary comprehensive hospitals with integrated clinical and teaching functions in Anhui and Jiangsu provinces, China. Jiangsu is an economically developed eastern coastal province, whereas Anhui is a central inland province; health-service supply and the distribution of the nursing workforce vary across regions and provinces in China. We selected hospitals from both provinces to increase contextual heterogeneity and reduce single-centre bias, while maintaining institutional comparability by restricting sites to tertiary grade A institutions.

### 2.2 Participants

Participants were recruited through convenience sampling. Inclusion criteria were: (1) possession of a national nurse practising licence; and (2) current employment in frontline clinical care or nursing management positions. Exclusion criteria were: (1) nurses on leave or otherwise off duty; and (2) nurses who declined to participate.

The required sample size for multivariable linear regression was estimated using the rule of at least 20 participants per predictor variable (20), and was inflated by 20% to account for potential invalid questionnaires, yielding a minimum target of 384. A total of 1,126 questionnaires were distributed, of which 925 were valid, yielding an effective response rate of 82.15%. To improve transparency regarding unit selection, questionnaires were distributed across a broad range of clinical departments/units in each participating hospital (e.g., internal medicine wards, surgical wards, emergency department, intensive care unit, and operating rooms), as coordinated by the nursing administration. Units were chosen to ensure coverage of major clinical service lines and to include both high-demand/high-risk and general inpatient settings, based on feasibility and unit managers’ collaboration. Within each selected unit, all eligible nurses were invited to participate.

### 2.3 Measures

#### 2.3.1 Sociodemographic questionnaire

A researcher-designed questionnaire was used to capture core characteristics—age, sex, position, professional title, educational attainment, and annual income—to characterise the sample and examine subgroup differences. These variables were also considered potential covariates in subsequent regression and mediation analyses because prior studies have shown that nurses’ safety behaviours and burnout may vary by demographic and job-related characteristics such as age, professional title, position, years of experience, employment type, and income (11–13, 21–23).

#### 2.3.2 Nurses’ safety behaviours questionnaire (NSBQ)

The NSBQ was originally developed by Shih et al. (8) and subsequently introduced and translated into Chinese by Rong (24). It comprises 12 items rated on a five-point Likert scale (1 = “never” to 5 = “always”). The total score ranges from 12 to 60, with higher scores indicating better safety behaviours. In this study, internal consistency was good (Cronbach’s *α* = 0.871).

#### 2.3.3 Maslach burnout inventory–general survey (MBI–GS, Chinese version)

Burnout was assessed using the Chinese version of the MBI–GS revised by Li and Shi (25), comprising three dimensions: exhaustion (5 items), cynicism (4 items), and professional efficacy (6 items). Items were rated on a seven-point Likert scale (0 = never to 6 = very frequently). Professional efficacy was reverse-scored so that higher total scores (range 0–90) indicate more severe burnout. Internal consistency in this study was good (Cronbach’s *α* = 0.882).

#### 2.3.4 Team climate inventory (TCI; short form)

Team climate was assessed using the short form of the TCI (26). The instrument comprises 14 items across four dimensions—vision (4 items), participative safety (4 items), task orientation (3 items), and support for innovation (3 items)—rated on a five-point Likert scale (1 = strongly disagree to 5 = strongly agree). Higher scores indicate a more positive team climate. Internal consistency in this study was good (Cronbach’s *α* = 0.883).

### 2.4 Data collection procedures

Prior to data collection, nurse managers at the three participating hospitals received standardised training from the research team and coordinated the on-site administration of questionnaires within their units. Within the selected units, recruitment followed a consecutive (all-eligible) approach, whereby all registered nurses meeting the inclusion criteria were invited during the survey period. After providing written informed consent, participants completed paper-based questionnaires within a designated time frame and returned them anonymously. Two trained researchers independently verified and double-entered the data. Questionnaires that were incomplete or exhibited obvious patterned responding were deemed invalid and excluded. No identifiable personal information was collected, and participation was voluntary.

### 2.5 Ethical considerations

This study was reviewed and approved by the Medical Ethics Committee of Anqing Municipal Hospital (Approval No. 2025-19; official ethics Document No. (2025) 19; effective date: March 7, 2025). Written informed consent was obtained from all participants, who participated voluntarily. Participants were informed that the data would be used solely for research purposes; responses were collected anonymously and handled in a de-identified manner, and personal information was kept strictly confidential. All procedures were conducted in accordance with relevant ethical guidelines and regulations and the Declaration of Helsinki.

### 2.6 Data analysis

**Figure 1:**
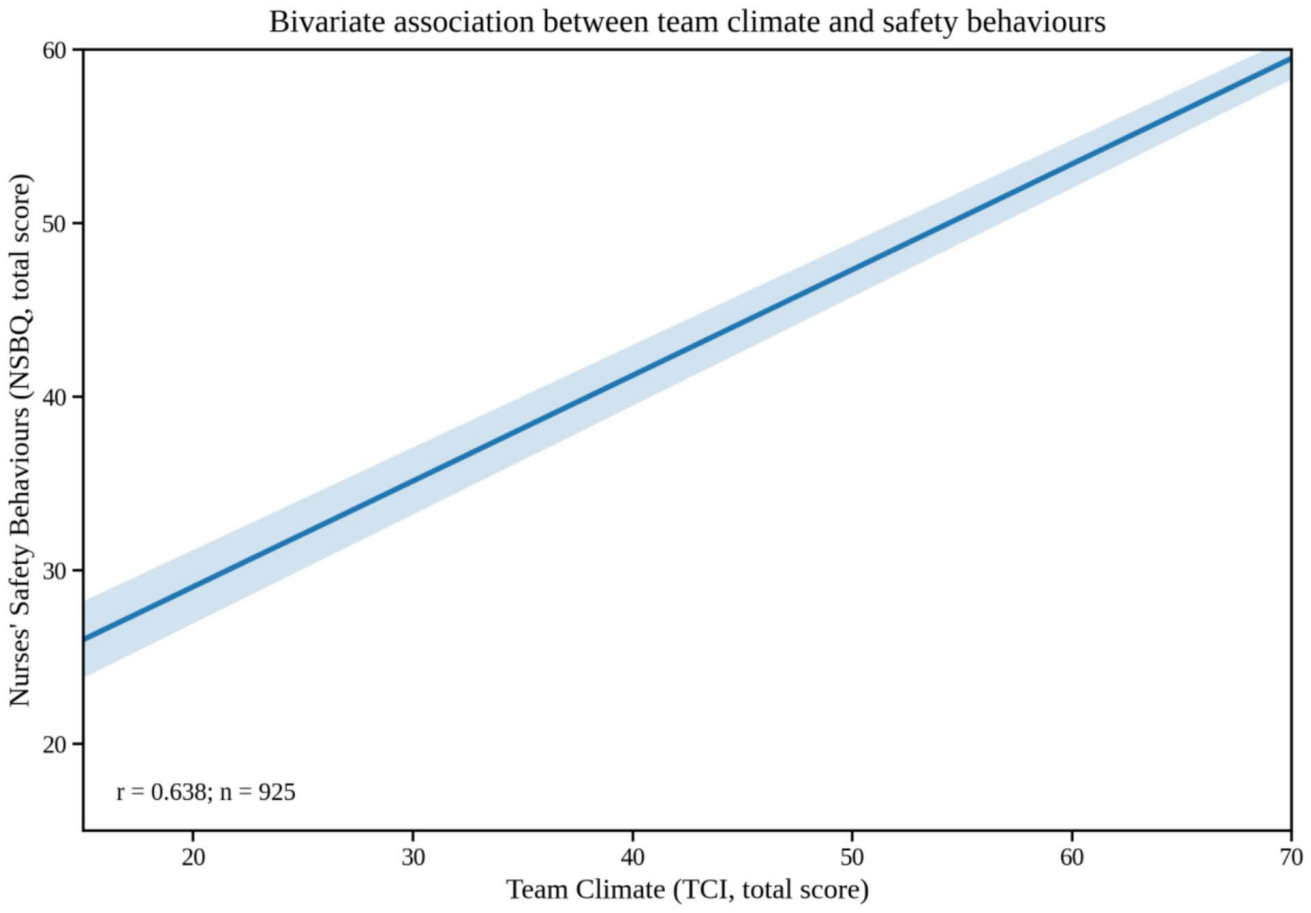
Team climate versus nurses’ safety behaviours (bivariate fitted regression line with 95% confidence band).

**Figure 2:**
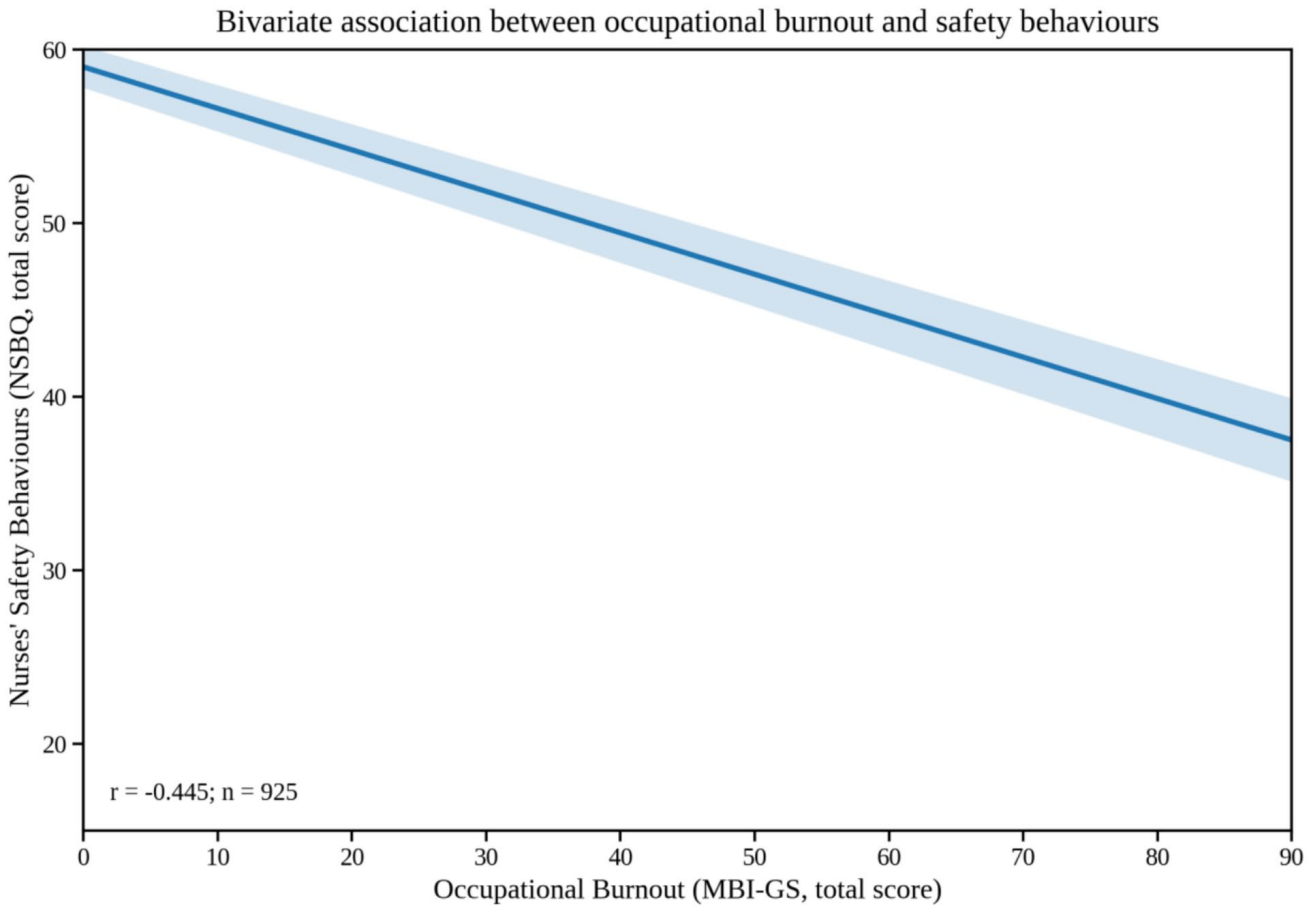
Occupational burnout versus nurses’ safety behaviours (bivariate fitted regression line with 95% confidence band).

All data were analysed using IBM SPSS Statistics (version 26.0). Continuous variables were expressed as mean ± standard deviation (mean ± SD), and categorical variables as counts and percentages. Independent-samples *t*-tests and one-way analysis of variance (ANOVA) were used to examine differences in safety behaviours, burnout, and team climate across sociodemographic subgroups. Pearson’s correlation analysis (Pearson’s *r*, two-tailed) was conducted to explore bivariate associations among the total and subscale scores of nurses’ safety behaviours (NSBQ), occupational burnout (overall and its three dimensions), and team climate (overall and its four dimensions). Correlations were computed using complete cases (listwise deletion), and statistical significance was set at *p* < 0.05 (two-tailed). For clarity, Table 1 reports correlation coefficients (*r*) with significance markers (**p < 0.01). To improve clarity and accessibility, we additionally visualized key bivariate relationships using fitted regression lines with 95% confidence bands (Figures 1, 2).

**Table 1:** Bivariate correlations among study variables (*N* = 925).

Variables		0	1	2	3	4	5	6	7	8	9
0	NSBQ	1									
1	Occupational burnout	−0.446**	1								
2	Exhaustion	−0.220**	0.739**	1							
3	Cynicism	−0.272**	0.765**	0.821**	1						
4	Professional efficacy	−0.392**	0.600**	−0.056	0.02	1					
5	Team climate	0.638**	−0.454**	−0.331**	−0.351**	−0.273**	1				
6	Vision	0.615**	−0.445**	−0.329**	−0.337**	−0.268**	0.936**	1			
7	Participative safety	0.618**	−0.448**	−0.327**	−0.350**	−0.267**	0.956**	0.854**	1		
8	Task orientation	0.565**	−0.369**	−0.264**	−0.287**	−0.226**	0.927**	0.806**	0.840**	1	
9	Support for innovation	0.605**	−0.443**	−0.321**	−0.345**	−0.267**	0.960**	0.851**	0.906**	0.892**	1

Pearson’s *r* (two-tailed). **p < 0.01. Higher burnout scores indicate more burnout; professional efficacy was reverse-scored.

Selection of covariates: Demographic and job-related variables were prespecified as potential confounders based on prior evidence that nurses’ safety behaviours and occupational burnout differ by age/tenure, professional rank, position, employment arrangement, and income, which may also be associated with perceptions of the team climate (11–13, 21–23). Therefore, age, professional title, position, years of experience, employment type, and annual income were included as covariates in the regression models. Sex and educational attainment were not entered as covariates because of the highly imbalanced distribution (e.g., 96.2% female) and the absence of significant subgroup differences in the outcome in preliminary analyses, which could otherwise reduce model stability.

Verification of regression assumptions: Prior to multivariable linear regression and mediation analyses, we verified key regression assumptions. Linearity was assessed by inspecting scatterplots and residuals-versus-fitted plots to ensure an approximately linear relationship between predictors and the outcome. Normality of residuals was evaluated using normal Q–Q plots of standardised residuals. Homoscedasticity was examined through residual plots and formal Breusch–Pagan tests. Potential influential observations were screened using leverage and Cook’s distance. Multicollinearity was assessed using variance inflation factors (VIFs) (and tolerances), with VIF < 5 indicating no concerning collinearity. Overall, diagnostic checks suggested no material violations of these assumptions (all VIFs < 5).

Sensitivity analyses were conducted by adding the prespecified demographic and job-related covariates (age, professional title, position, years of experience, employment type, and annual income) into the mediation models to examine the robustness of the indirect effects.

Multivariable linear regression and mediation analyses were performed using the PROCESS macro (Model 4) for SPSS (27). A bootstrap procedure with 5,000 resamples was used to estimate bias-corrected 95% confidence intervals (CIs). Mediation effects were deemed statistically significant when the 95% CI excluded zero. Statistical significance was set at two-tailed *p* < 0.05.

## 3 Results

### 3.1 Common method bias test

Given that the data were primarily derived from self-reported questionnaires, common method bias (CMB) was assessed. Following Harman’s single-factor test, an unrotated principal component analysis (PCA) showed that the first factor accounted for 36.33% of the total variance, below the commonly used 40% threshold. This result suggests that CMB was not a significant concern (28). However, Harman’s single-factor test is a coarse diagnostic and cannot fully rule out common method variance; thus the findings should be interpreted with appropriate caution (28). Therefore, the data were considered suitable for subsequent analyses, including mediation models.

### 3.2 Demographic characteristics of participants

A total of 925 valid questionnaires were included, yielding an effective response rate of 82.15%. Among the participants, 96.2% were female, and 74.7% held a bachelor’s degree. Detailed demographic characteristics are shown in Table 2.

**Table 2:** Sociodemographic differences in nurses’ safety behaviours (*N* = 925).

Variables	*n*	%	Nurse safety behaviour	*t/F*
			M ± SD	
Gender				0.716
Female	890	96.2	52.9 ± 9.18	
Male	35	3.8	51.8 ± 8.32	
Age (in years)				6.64*
18–25	133	14.4	50.6 ± 9.39	
26–35	442	47.8	52.3 ± 9.62	
35–45	255	27.6	54.1 ± 8.59	
45–55	93	10.1	55.9 ± 6.46	
≥56	2	0.2	50 ± 14.14	
Education				1.03
College degree or below	225	24.3	52.7 ± 8.58	
Undergraduate	691	74.7	53 ± 9.31	
Postgraduate	9	1	48.4 ± 9.86	
Professional title				5.054**
Junior nurse or below	487	52.6	52.1 ± 9.36	
Senior nurse	357	38.6	53.5 ± 8.92	
Associate superintendent nurse	81	8.8	55 ± 8.37	
Position				16.7***
No title	816	88.2	52.7 ± 9.14	
Head nurse	103	11.1	54.4 ± 9.21	
Head of the nursing department	6	0.7	58.5 ± 2.35	
Work experience (year)				7.64***
≤5	222	24	51.9 ± 8.57	
6–10	225	24.3	51.3 ± 10.42	
11–20	331	35.8	53.6 ± 9.09	
≥21	147	15.9	55.1 ± 7.3	
Form of appointment				4.595*
Temporary employment	211	22.8	52.5 ± 8.39	
Permanent staff	242	26.2	54.3 ± 8.3	
Contract employee	472	51	52.3 ± 9.8	
Annual income (RMB)				5.79**
≤100,000	632	68.3	52.2 ± 9.45	
110,000–150,000	249	26.9	54.2 ± 8.36	
≥160,000	44	4.8	54.9 ± 8	

*p < 0.05. **p < 0.01. ***p < 0.001.

Group comparisons (independent-samples *t*-tests/one-way ANOVA, as appropriate) showed that nurses’ safety behaviours differed significantly by age (*F* = 6.64, *p* < 0.05), professional title (*F* = 5.05, *p* < 0.01), position (*F* = 16.70, *p* < 0.001), work experience (*F* = 7.64, p < 0.001), Form of appointment (*F* = 4.595, *p* < 0.05), and annual income (*F* = 5.79, *p* < 0.01).

### 3.3 Scores of nurses’ safety behaviours, burnout, and team climate

As shown in Table 3, the mean score for nurses’ safety behaviours was 52.89 ± 9.14, indicating a relatively high level of safety behaviours. The mean burnout score was 26.84 ± 16.94, reflecting a moderate-to-high level of burnout. The mean team climate score was 59.31 ± 9.74, suggesting a generally favourable team climate.

**Table 3:** Descriptive statistics for safety behaviours, occupational burnout, and team climate scores.

Subscale	Item	Score
NSBQ	12	52.89 ± 9.14
Occupational burnout	15	26.84 ± 16.94
Exhaustion	5	9.94 ± 8.09
Cynicism	4	5.41 ± 3.10
Professional efficacy	6	11.49 ± 10.49
Team climate	14	59.31 ± 9.74
Vision	4	16.78 ± 2.91
Participative safety	4	17.2 ± 2.94
Task orientation	3	12.55 ± 2.23
Support for innovation	3	12.78 ± 2.23

### 3.4 Correlations among nurses’ safety behaviours, burnout, and team climate

Table 1 presents Pearson’s bivariate correlations (two-tailed) among the main study variables (*N* = 925). Because professional efficacy in the MBI–GS was reverse-scored, higher scores indicate reduced professional efficacy (i.e., more severe burnout), and correlations were interpreted accordingly. As shown in Table 1, nurses’ safety behaviours were negatively correlated with the total burnout score (*r* = −0.445, *p* < 0.01) and with its three dimensions—emotional exhaustion (*r* = −0.220), cynicism (*r* = −0.272), and reduced personal accomplishment (*r* = −0.392) (all *p* < 0.01). By contrast, safety behaviours were positively correlated with the total team climate score (*r* = 0.638) and with its four dimensions—vision (*r* = 0.615), participative safety (*r* = 0.618), task orientation (*r* = 0.565), and support for innovation (*r* = 0.605) (all *p* < 0.01). In addition, team climate was negatively correlated with burnout (*r* = −0.454, *p* < 0.01). For visual clarity, Figure 1 illustrates the positive association between team climate and nurses’ safety behaviours, and Figure 2 illustrates the negative association between occupational burnout and nurses’ safety behaviours.

### 3.5 Regression and mediation analysis

#### 3.5.1 Hierarchical regression analysis

Nurses’ safety behaviours was set as the dependent variable. In Model 1, we entered the prespecified demographic and job-related covariates (age, professional title, position, years of experience, employment type, and annual income) to adjust for potential confounding based on prior evidence (11–13). Results indicated that only age predicted safety behaviours (*B* = 1.776, *p* = 0.006), explaining 2.9% of the variance (*R*^2^ = 0.029).

In Model 2, team climate and burnout were added as additional predictors. Burnout negatively predicted safety behaviours (*B* = −0.101, *β* = −0.188, 95% CI − 0.131 to −0.072, *p* < 0.001), whereas team climate positively predicted them (*B* = 0.518, *β* = 0.551, 95% CI 0.467 to 0.569, *p* < 0.001). Model fit improved from *R*^2^ = 0.029 to *R*^2^ = 0.456 (Δ*R*^2^ = 0.427). Detailed results are presented in Table 4.

**Table 4:** Regression analysis of nurses’ safety behaviours.

Variables	Model 1					Model 2				
	*B*	*β*	*p*	95%CI	VIF	*B*	*β*	*p*	95%CI	VIF
(constant)	48.237		*p* < 0.001	45.285 to 51.19		22.096		*p* < 0.001	17.945 to 26.247	
Age	1.776	0.165	0.006	0.515 to 3.037	3.371	1.028	0.096	0.033	0.081 to 1.975	3.383
Professional title	−0.487	−0.035	0.486	−1.856 to 0.883	2.322	0.138	0.01	0.792	−0.891 to1.167	2.333
Position	0.57	0.022	0.555	−1.325 to 2.465	1.286	0.998	0.038	0.169	−0.424 to 2.419	1.288
Years of experience	−0.092	−0.01	0.867	−1.171 to 0.987	3.569	0.147	0.016	0.721	−0.663 to 0.957	3.583
Employment type	−0.103	−0.009	0.781	−0.828 to 0.622	1.017	−0.384	−0.034	0.168	−0.93 to 0.162	1.026
Annual income	0.787	0.049	0.201	−0.42 to 1.995	1.399	−0.314	−0.02	0.499	−1.223 to 0.596	1.414
Occupational burnout						−0.101	−0.188	P < 0.001	−0.131 to-0.072	1.29
Team climate						0.518	0.551	P < 0.001	0.467 to 0.569	1.282
*R*^2^/adjusted *R*^2^	0.029/0.023					0.456/0.451				
Δ*R*^2^	0.427									

*B*, unstandardised coefficient; *β*, standardised coefficient; CI, 95% confidence interval; VIF, variance inflation factor.

#### 3.5.2 Mediation analysis

To test the mediating role of burnout, we further analysed the hierarchical regression results using Hayes’s PROCESS macro (Model 4) for SPSS (27), with bootstrapping (5,000 resamples) to obtain bias-corrected 95% CIs. Mediation effects were considered statistically significant when the 95% CI excluded zero.

**Figure 3:**
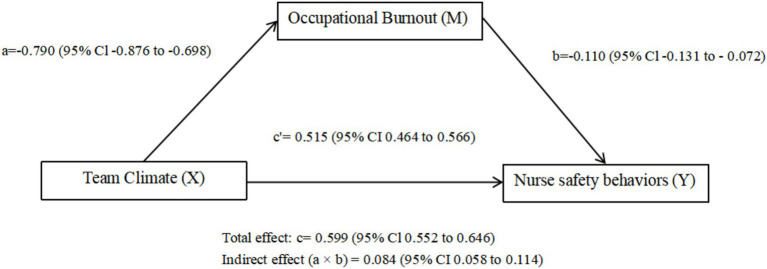
Total effect model and mediation pathway. Unstandardised coefficients (*B*) and 95% confidence intervals (CIs) are annotated on the diagram. Path *a* = effect of X on M; *b* = effect of M on Y; *c′* = direct effect of *X* on *Y*; *c* = total effect. Indirect effect is *a* × *b*. *N* = 925.

Results indicated that burnout mediated the relationship between team climate and nurses’ safety behaviours. The total effect of team climate on safety behaviours was *B* = 0.599 (95% CI: 0.552–0.646), the direct effect was *B* = 0.515 (95% CI: 0.464–0.566), and the indirect effect through burnout was *B* = 0.084 (95% CI: 0.058–0.114). Because the CI for the indirect effect did not include zero, the mediating effect of burnout was statistically significant. These findings support Hypothesis H2, suggesting that burnout partially mediates the association between team climate and nurses’ safety behaviours. Detailed results are presented in Table 5 and illustrated in Figure 3 with path coefficients and 95% CIs.

**Table 5:** Results of mediation analysis.

Class	*B*	SE	LLCI	ULCI	Quantity of effect
Total effect	0.599	0.024	0.552	0.646	
Direct effect	0.515	0.026	0.464	0.566	86.0%
Indirect effect	0.084	0.014	0.058	0.114	14.0%

*B*, unstandardised coefficient; SE, standard error; LLCI/ULCI, lower/upper limit of 95% confidence interval.

## 4 Discussion

Grounded in the JD–R model, this study explored the associations among team climate, occupational burnout, and nurses’ safety behaviours. Given the cross-sectional design, the observed relationships should be interpreted as correlational and not as evidence of causality. We found that nurses’ safety behaviours were above average. Team climate was positively associated with safety behaviours, whereas occupational burnout was negatively associated with them. Mediation analyses further indicated a statistically significant indirect association via burnout that was consistent with partial mediation, suggesting that burnout may represent one potential pathway linking team climate to safety behaviours.

### 4.1 Current status of nurses’ safety behaviours and demographic differences

In this study, the mean score for nurses’ safety behaviours was 52.89 ± 9.14, indicating an above-average level. This finding is generally consistent with those reported by Lee et al. (29) and Ma et al. (30). In recent years, China’s policies on healthcare quality and patient safety have been progressively refined, accompanied by the ongoing optimisation of hospital management systems. These developments have provided a supportive institutional and environmental framework for improving nurses’ safety behaviours (31). In particular, within large tertiary general hospitals, well-established management structures, advanced infrastructure, and highly qualified nursing teams collectively foster an environment conducive to safe nursing practice (32).

Further analysis revealed significant differences in safety behaviours scores by age, professional title, position, years of experience, employment status, and annual income. Nurses who were older, held higher professional titles, occupied managerial roles, had ≥ 21 years of experience, held permanent employment, and reported higher annual income exhibited higher safety behaviours scores. These findings align with those reported by Qi et al. (21) and Zhang et al. (22).

Senior nurses typically possess more clinical experience and stronger risk-recognition abilities, while individuals with advanced professional titles or managerial roles tend to exhibit a greater sense of professional norms and responsibility, thereby showing greater adherence to safety practices (21, 23). Moreover, stable employment and adequate financial compensation contribute to a stronger professional identity and work engagement, which, in turn, facilitate the consistent enactment of safety behaviours (23).

These findings highlight the need to optimise nursing team composition and strengthen career-development support within nursing management. Targeted training and incentive programmes should be tailored to the diverse demographic and professional profiles of nurses, thereby promoting sustained improvements in safety performance.

### 4.2 Direct promoting effect of team climate on nurses’ safety behaviours

This study supported Hypothesis H1, indicating that team climate was positively associated with nurses’ safety behaviours. Within the JD–R framework, team climate can be conceptualised as a job resource; a more favourable team climate may be linked to higher work engagement and stronger motivation to comply with safety practices, which may, in turn, relate to better safety behaviours (33).

A positive team climate may signal organisational support and recognition, potentially strengthening nurses’ sense of belonging and professional identity, which may, in turn, encourage adherence to safety standards and participation in safety-improvement initiatives (34–36). Evidence suggests that a favourable team climate is associated with greater psychological safety, higher communication quality, and increased efficiency and willingness to collaborate among team members (37, 38).

Moreover, when psychological safety is high, nurses are more inclined to communicate openly, which may help reduce nursing errors, enhance job satisfaction, decrease turnover intention, and potentially promote patient safety (39). In a non-punitive, inclusive team environment, nurses are more likely to proactively report potential safety risks and take preventive measures, potentially reducing the likelihood of AEs (40).

In this study, the task-orientation and innovation-support dimensions of team climate received relatively high scores. These dimensions provide clear behavioural guidance and opportunities for improvement, offering a set of positive behavioural norms internalised by team members. Such a climate fosters knowledge exchange and collaborative learning, thereby promoting the continuous refinement of safety practices (41).

In addition, Tao et al. (42) reported that a favourable team climate enhances perceived organisational support and organisational commitment, exerting a strong influence on knowledge-sharing behaviours. Knowledge sharing enables nurses to acquire up-to-date clinical techniques and best practices, strengthening safety awareness and emergency-response capacity, while peer exchange consolidates team learning, reduces operational errors, and elevates overall safety behaviours within units.

In a positive team climate, nurses tend to cultivate a shared culture of safety, in which normative behaviours are reinforced through peer modelling and mutual encouragement (43, 44).

Building on the subgroup comparisons, nurses’ safety behaviour scores differed by work experience and position (Table 2): nurses with ≤10 years of experience reported lower scores (≤5 years: 51.9 ± 8.57; 6–10 years: 51.3 ± 10.42) than those with ≥21 years of experience (55.1 ± 7.30), and scores were higher among managerial nurses (head nurse: 54.4 ± 9.21; head of the nursing department: 58.5 ± 2.35) than those without a management title (52.7 ± 9.14). These patterns suggest that strengthening team climate may be particularly beneficial for early-career and non-managerial nurses by increasing supervision quality, feedback, and communication-supportive practices. Future research should examine whether these patterns vary by unit type, which was not stratified in the current analyses.

### 4.3 The mediating role of occupational burnout

This study supported the indirect effect of occupational burnout in the association between team climate and nurses’ safety behaviours, which was consistent with partial mediation and thereby aligned with H2. This is consistent with the JD–R model, which suggests that job resources may buffer stress and potentially reduce burnout, thereby lessening its detrimental association with safety behaviours (19).

The negative association between team climate and burnout aligns with findings from a multicentre study in Greece reporting a close relationship between the work environment and nurses’ burnout levels (45). This suggests that cultivating a supportive, resource-enriched team climate may help reduce burnout and sustain engagement in safety practices.

Nevertheless, although the mediating effect of burnout was significant, its magnitude was modest. In particular, the indirect effect (*B* = 0.084) accounted for approximately 14.0% of the total effect (*B* = 0.599) (Table 5), suggesting that burnout explains only a small portion of how team climate translates into safety behaviours. Practically, this pattern indicates that interventions aimed at improving team climate may yield the largest gains through direct motivational and normative pathways, whereas burnout mitigation remains a complementary—rather than dominant—lever for strengthening safety behaviours. Two mechanisms may explain this finding. First, in nursing—a profession characterised by high and sustained job demands—the sources of work stress are multifaceted and continuous. According to Conservation of Resources (COR) theory, employees’ sustained cognitive and physical exertion in high-demand settings increases strain, leading to psychological resource depletion and a greater need for recovery (46). Under such conditions, a single job resource—such as team climate—may be insufficient to offset the impact of prolonged demands.

Second, the development of occupational burnout is a chronic, multifactorial process (47), influenced by organisational factors as well as individual psychological resilience, well-being, and workload intensity. Thus, even within a favourable team climate, underlying antecedents of burnout may persist. Prior research has identified environmental demands and personal resources as critical antecedents of nurse burnout (46). Once burnout emerges, even in supportive teams, nurses may experience fatigue and emotional exhaustion, resulting in reduced energy and motivation to sustain safety behaviours—particularly under high workload or in emergencies.

In line with COR theory, emotional exhaustion and depersonalisation—the core dimensions of burnout—indicate severe depletion of psychological resources. For nurses scoring high on these dimensions, internal resources are likely depleted, rendering them less able to translate external support into the cognitive and emotional resources necessary to enact safety behaviours (48). This effect is especially pronounced among individuals with lower psychological resilience, for whom symptoms of emotional exhaustion and depersonalisation can become overwhelming and debilitating (49).

Practical note. While detailed managerial implications are presented elsewhere in this manuscript, our findings reinforce the importance of balancing resource allocation and demand management, cultivating a positive team climate, and providing recovery opportunities and restorative training (e.g., mindfulness-based or relaxation training) to sustain nurses’ long-term professional vitality and engagement in safety behaviours.

## 5 Limitations

First, this study employed a cross-sectional design, which, although useful for identifying correlations and mediating pathways among variables, does not allow for causal inference about the relationships between team climate, occupational burnout, and safety behaviours. Future studies could employ experimental or longitudinal designs to more rigorously test the causal mechanisms among these variables.

Second, the sample was drawn from three large tertiary hospitals in Anhui and Jiangsu provinces, China. Although the sample size was relatively large, the use of convenience sampling may have introduced selection bias, limiting the representativeness and generalisability of the findings. Future research should consider multiregional and multicentre probability sampling to improve external validity. In addition, although we distributed questionnaires across multiple clinical units to improve coverage, departments/units were not randomly selected and analyses were not stratified by unit type, which may limit representativeness at the unit level.

Third, all key variables were measured using self-administered instruments (NSBQ, MBI-GS, and TCI), which may introduce self-report bias. Specifically, NSBQ responses may be influenced by social desirability and recall bias, leading to overreporting of desirable safety behaviours; MBI-GS scores may be affected by stigma or evaluation concerns, resulting in underreporting of burnout symptoms; and TCI ratings may reflect transient mood states or respondents’ general affect rather than stable team-level characteristics. In addition, measuring predictors, mediator, and outcome using the same method at the same time point may contribute to common method variance and potentially inflate the estimated associations. Although we adopted procedural remedies (anonymous completion, confidentiality assurance, and standardised instructions) and assessed common method bias using Harman’s single-factor test, such approaches cannot completely rule out method effects (28, 50, 51). Future studies should triangulate self-reports with objective or multi-source indicators (e.g., incident reports, observational audits, supervisor ratings, or peer assessments) and consider time-lagged/longitudinal designs to reduce bias and strengthen causal inference.

Fourth, while this study controlled for several demographic variables, unmeasured confounding factors may remain, including unit/department type, hospital level, and workload intensity, all of which may influence nurses’ safety behaviours and occupational burnout. Future studies should incorporate a broader range of organisational and individual-level variables to provide a more comprehensive understanding of the underlying mechanisms.

### 5.1 Implications for nursing management

The findings can inform more actionable nursing-management strategies by adopting a tiered approach tailored to nurses’ career stage and clinical context. At the organisational level, managers may first conduct unit-level monitoring of safety behaviours and burnout (e.g., periodic screening and feedback) and prioritise high-demand/high-risk units (e.g., emergency, intensive care, operating rooms) for targeted resource allocation, staffing optimisation, and workflow standardisation.

For early-career nurses (e.g., ≤10 years of experience) and non-managerial staff—groups that reported comparatively lower safety behaviour scores in this study—interventions may emphasise structured onboarding and competency development. Practical actions include formal preceptorship/mentoring, simulation-based training for high-risk procedures, checklists and standard operating procedures for key safety practices, and regular safety huddles with non-punitive feedback to strengthen psychological safety and speaking-up behaviours.

For more experienced nurses and those in leadership roles, interventions may focus on leveraging their expertise to reinforce team norms and sustain improvement. Nurse leaders may appoint safety champions, embed peer coaching and reflective debriefing into routine practice, and involve senior staff in quality-improvement cycles (audit and feedback, incident learning, and unit-based problem solving). Where feasible, unit-specific adaptations (e.g., enhanced handover protocols and rapid debriefs in emergency/ICU settings) may help align team climate initiatives with local workflow demands.

Because burnout showed a significant (albeit modest) indirect association, burnout-prevention efforts should be integrated with team-climate initiatives rather than delivered as standalone programmes. Actionable options include flexible scheduling, protected breaks and recovery opportunities, access to psychological support, and skills-based stress-management training (e.g., mindfulness or relaxation). Tailoring intensity to unit workload and individual risk (e.g., transition support for newly employed nurses; additional recovery resources in high-demand units) may help maintain sustained engagement in safety practices.

## 6 Conclusion

This study provides empirical support for the JD–R framework in explaining nurses’ safety behaviours. Overall, the findings suggest that team climate is positively associated with safety behaviours and that occupational burnout is negatively associated with them. The mediation results further suggest that lower burnout may represent one potential pathway through which team climate is related to safety behaviours, although causal inferences cannot be made from the current cross-sectional design.

Moreover, the indirect effect via burnout, although statistically significant, was modest in magnitude (indirect effect = 0.084, accounting for 14.0% of the total effect). This suggests that the association between team climate and safety behaviours may operate largely through direct pathways, and that other organisational and individual factors—such as workload intensity, psychological resilience, and perceived organisational support—may also contribute to nurses’ engagement in safety practices.

Overall, this research enriches the theoretical understanding of nursing safety behaviours by integrating insights from the JD–R and COR frameworks. Practically, it underscores the need for hospital management to create a supportive team climate, optimise work-resource allocation, and implement burnout-prevention strategies to sustain nurses’ well-being and enhance patient safety. Future studies employing longitudinal or experimental designs and multicentre probability sampling are recommended to further validate and generalise these findings.

## Data Availability

The original contributions presented in the study are included in the article/supplementary material, further inquiries can be directed to the corresponding authors.
